# Human cell dedifferentiation in mesenchymal condensates through controlled autophagy

**DOI:** 10.1038/srep13113

**Published:** 2015-08-20

**Authors:** Rebecca Pennock, Elen Bray, Paul Pryor, Sally James, Paul McKeegan, Roger Sturmey, Paul Genever

**Affiliations:** 1 Department of Biology, University of York, York, YO10 5DD, UK; 2 Centre for Cardiovascular and Metabolic Research, Hull York Medical School, University of Hull, Hull, HU6 7RX, UK

## Abstract

Tissue and whole organ regeneration is a dramatic biological response to injury that occurs across different plant and animal phyla. It frequently requires the dedifferentiation of mature cells to a condensed mesenchymal blastema, from which replacement tissues develop. Human somatic cells cannot regenerate in this way and differentiation is considered irreversible under normal developmental conditions. Here, we sought to establish *in vitro* conditions to mimic blastema formation by generating different three-dimensional (3D) condensates of human mesenchymal stromal cells (MSCs). We identified specific 3D growth environments that were sufficient to dedifferentiate aged human MSCs to an early mesendoderm-like state with reversal of age-associated cell hypertrophy and restoration of organized tissue regenerating capacity *in vivo*. An optimal auophagic response was required to promote cytoplasmic remodeling, mitochondrial regression, and a bioenergetic shift from oxidative phosphorylation to anaerobic metabolism. Our evidence suggests that human cell dedifferentiation can be achieved through autonomously controlled autophagic flux.

Dedifferentiation of mature cells enables epimorphic regeneration of lost tissues and organs in some lower organisms such as teleost fish and amphibians. Examples include caudal fin regeneration in zebrafish, tail regeneration in *Xenopus* tadpoles and whole limb regeneration in adult salamanders^1^,^2^. Despite clear structural and anatomical differences, a common prerequisite for tissue regeneration is the formation of a blastema; a condensation of dedifferentiated mesenchymal cells from which the regenerated tissue arises^1^. In mammals, particularly humans, regeneration is highly restricted and differentiation during embryonic and postnatal development is normally considered to be irreversible, although recent work has demonstrated the capacity for dedifferentiation in some mammalian cells following tissue ablation or injury^3^,^4^,^5^. Human somatic cells can be artificially induced to dedifferentiate by introducing defined factors such as Oct4 (also known as POU5F1), Klf4, Sox2 and cMyc^6^ or Oct4, Sox2, Nanog and Lin28^7^ to generate induced pluripotent stem cells (iPSCs), which have embryonic stem cell (ESC)-like characteristics. Direct reprogramming protocols aim to convert (transdifferentiate) one mature cell type to another, circumventing pluripotency and teratoma risk^8^,^9^,^10^. Lineage-restricted factor-based reprogramming can dedifferentiate cells to non-tumorigenic, developmentally primitive precursors, and can be achieved *in vivo*^11^ offering an attractive clinical option for tissue regeneration. However, almost all reprogramming strategies have focused on nuclear reprogramming using non-physiological conditions to drive dedifferentiation. Recent comparative studies demonstrated that homologues of iPSC pluripotent factors are induced in blastemal cells during appendage regeneration in zebrafish, *Xenopus* and newts^12^,^13^, suggesting that similar molecular mechanisms operate during non-physiological human cell dedifferentiation (e.g. in iPSC generation) and natural dedifferentiation in lower organisms. Here, we hypothesised that rudimentary blastema-like conditions could be established in condensates of human bone marrow mesenchymal stromal cells (MSCs) to induce dedifferentiation under physiological conditions. Human MSCs are a heterogeneous population of stromal cells, a fraction of which will be stem cells capable of generating bone, cartilage and fat tissues and therefore MSCs have broad similarities to mesenchymal cells in regenerative blastemas^14^. In addition, MSCs can regulate the haematopoietic marrow compartment through the secretion of the cytokine CXCL12 (also known as SDF-1) which interacts with the receptor CXCR4 expressed by haematopoietic stem cells^15^. Using varied three-dimensional (3D) *in vitro* models we initially examined Oct4, Nanog and Sox2 levels in different 3D conditions to screen for evidence of dedifferentiation. This approach identified specific conditions to induce efficient lineage-restricted dedifferentiation to a non-pluripotent, early mesendoderm-like state, switching from a CXCL12^High^ CXCR4^–^ to a CXCL12^Low^ CXCR4^+^ Brachyury^+^ population, mediated by metabolic conversion and a controlled autonomous autophagic response. Importantly, the dedifferentiated MSCs were capable of generating histologically-organised mesodermal structures when implanted *in vivo*; a property lacked by the parent non-dedifferentiated MSC population grown in standard 2D conditions. Our findings demonstrate an intrinsic capacity for human cell dedifferentiation that may have fundamental implications in regenerative biology.

## Results

### Optimized 3D Culture Induces Mesenchymal Condensation and Expression of Dedifferen-tiation Markers

**Figure 1:**
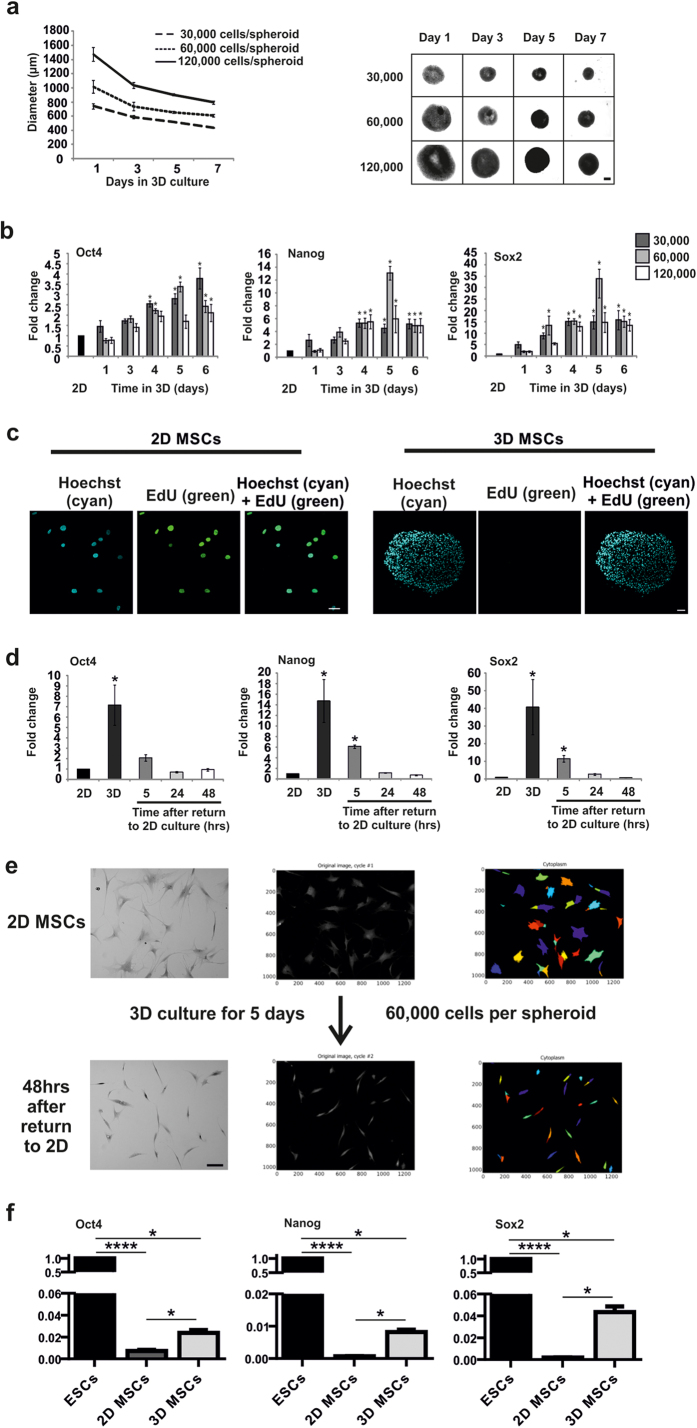
Dedifferentiation screening in 3D MSC condensates. (**a**) Analysis of 3D MSC formation and size over 7 days in culture. Left panel, diameter mean values shown ± SD, n = 6; right panel micrographs of whole spheroids, (scale bar = 250 μm). 30, 000, 60, 000 and 120, 000 refer to initiating cell number. (**b**) Expression of *Oct4, Nanog* and *Sox2* determined by quantitative polymerase chain reaction (QPCR) in different sized spheroids over time in 3D culture. Data represent three separate experimental donors (n = 3) and are shown as mean values ± SEM, * = p < 0.05. 30, 000, 60, 000 and 120, 000 refer to initiating cell number. (**c**) Immunofluorescent detection of EdU incorporation in 3D MSCs to identify proliferating cells (2D MSCs are shown as a positive control), scale bar = 50 μm. (**d**) QPCR analysis of pluripotency factors following disaggregation of 3D MSCs and return to 2D culture. Data represent three separate experimental donors (n = 3) and are shown as mean values ± SEM, * = p < 0.05. (**e**) Effects of 3D culture on morphology and size of MSCs that had been *in vitro*-aged to induce senescence-associated hypertrophy through extended time in culture. Brightfield image (left), darkfield image (middle) with arbitrary colour mask using CellProfiler software (right) to aid visualiation (scale bar = 100 μm). (**f**) QPCR analysis of Oct4, Nanog and Sox2 in 3D MSCs and 2D MSCs compared to ESCs. Data represent three separate experimental donors (n = 3) and are shown as mean values ± SEM, * = p < 0.05, **** = p < 0.0001. See also Figure S1.

We isolated MSCs from aged donors (range 56–79 years, median = 66.5, n = 12) based on plastic-adherence. We established a range of 3D spheroid *in vitro* growth conditions to examine the effects on MSC behaviour. 3D MSCs, grown at different initial cell numbers (30,000–120,000 cells/spheroid) for up to 7 days in culture, condensed to decrease in size over time (Fig. 1a), and efficiently established a wide repertoire of spheroid sizes to screen for evidence of dedifferentiation. We found that expression of pluripotent transcription factors, *Oct4, Nanog* and *Sox2*, increased significantly in 3D MSCs compared to 2D MSCs by quantitative polymerase chain reaction (QPCR). Considering the potential size/compositional variations in heterogeneous primary MSC populations, we identified a remarkably consistent and significant increase in mRNA expression for all pluripotent factors between days 3 to 5 of culture across donors with levels declining by day 6. These findings were most consistently replicated when 60,000 MSCs were used to establish the spheroid (Fig. 1b), therefore this culture model (day 5, 60,000 initiating cells) was used in subsequent experiments. The culture of MSCs as 3D condensates did not result in significant cell death (Supplementary Fig. S1). PI staining of 3D MSCs at days 1, 3 and 5 revealed that the percentage of dead cells in these cultures was 1.6% ± 0.4%, 3.6% ± 0.4% and 2.1% ± 0.6%, respectively (mean ± SEM, n = 4). The absence of EdU incorporation (Fig. 1c) and Ki67 staining (Supplementary Fig. S1) demonstrated that MSCs did not proliferate as 3D condensates, unlike the normal proliferative activity of MSCs in standard 2D culture. Therefore 3D culture conditions did not select for the survival and expansion of a pre-existing primitive cell type in a mixed initiating MSC population. Enhanced expression of pluripotency factors in 3D MSCs was transient and dependent on 3D culture. Following disaggregation and re-plating onto tissue culture plastic, expression levels of *Oct4, Nanog* and *Sox2* decreased significantly within 5 hours and returned to monolayer levels by 24–48 hours (Fig. 1d). Notably, the disaggregated cells had a markedly reduced cell area compared to 2D MSCs prior to 3D culture. This was particularly striking following *in vitro* ageing by repeat passaging until population doublings reached a plataeu to induce senescence-associated hypertrophy (average cell area = 8180 ± 2750 μm^2^ and 1240 ± 410 μm^2^ before and after 3D culture respectively, p < 0.0001, Fig. 1e), indicating that cell size shrinkage is a component of 3D condensation. By direct comparison with ESCs, we found that mRNA expression of *Oct4, Nanog* and *Sox2* in 3D MSCs did not reach levels expressed by pluripotent cells (Fig. 1f), similar to the low level mRNA expression of pluripotency factors observed in blastema cells in regenerating *Xenopus*, zebrafish and newts^12^,^13^. We were also unable to detect protein expression of pluripotency factors in 3D MSCs, by flow cytometry and immunocytochemistry (data not shown). Therefore 3D MSCs, like regenerating blastemas, do not reprogramme to full pluripotency but may also undergo restricted lineage dedifferentiation.

### Efficient Dedifferentiation to Early Mesendodermal, Tissue-Regenerating Progenitor Cells in 3D Mesenchymal Condensates

**Figure 2:**
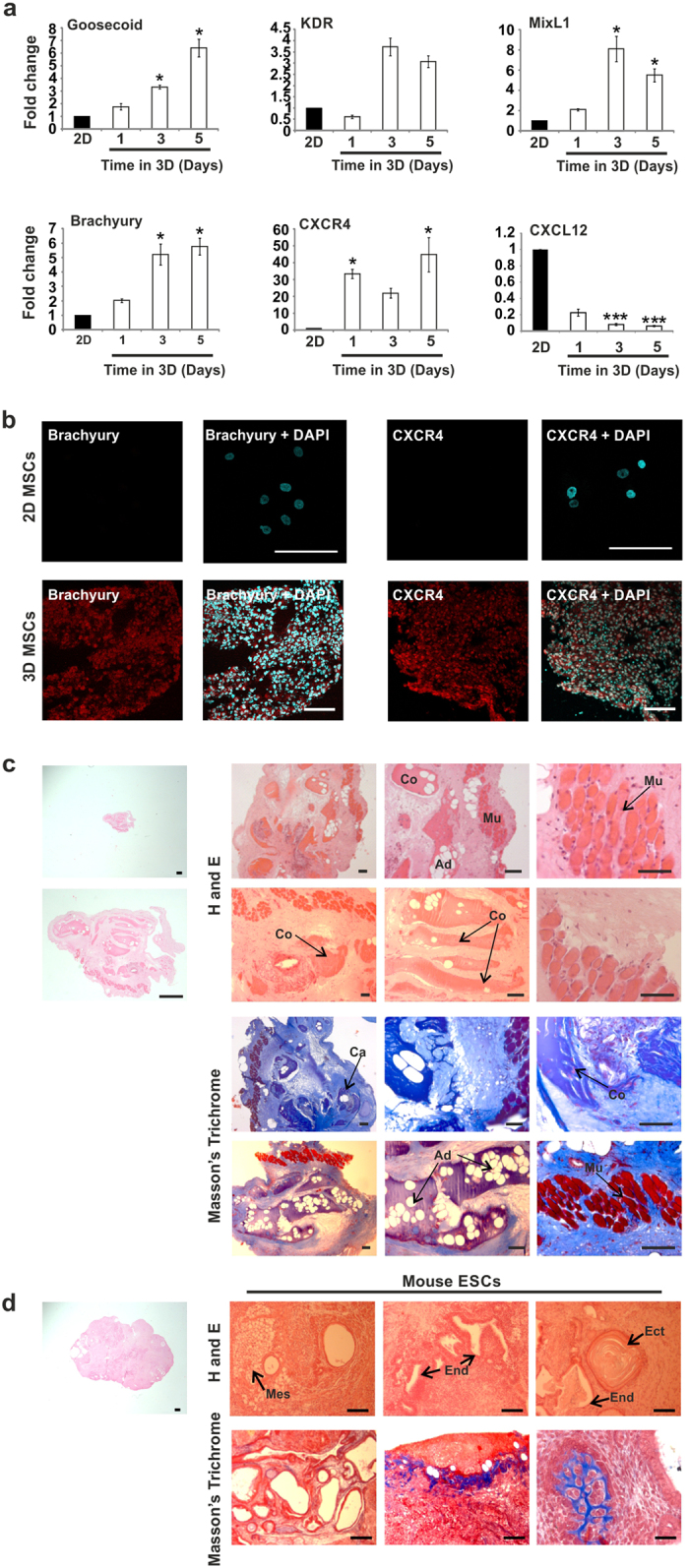
Dedifferentiation status and *in vivo* potency of 3D MSCs. (**a**) QPCR analysis of markers of early mesendoderm in 3D MSCs (expression normalized to GAPDH, fold changes calculated as 2(-DDCt)) compared to 2D MSCs. Data represent two separate experimental donors (n = 2) and are shown as mean values ± SEM, * = p < 0.05, *** = p< 0.001. (**b**) Immunofluorescent detection (red fluorescence) of Brachyury and CXCR4 expression in 2D MSCs (top panel) 3D MSC condensates (bottom panel) (scale bar = 100 μm). Nuclei identified by DAPI staining (blue fluorescence). (**c**) *In vivo* tissue formation assay using 3D MSC condensates, tissues labelled as muscle (Mu), cartilage (Ca), adipose (Ad) and connective tissue (Co). Sections stained with hematoxylin and eosin (H and E) and Masson’s Trichrome. All micrographs show tissues formed by 3D MSC condensates. Scale bars = 500 μm (far left panel only) and 100 μm. (**d**) *In vivo* tissue formation assay using mouse ESCs, germ layers identified as Mesoderm (Mes), Endoderm (End) and Ectoderm (Ect), sections stained with hematoxylin and eosin (**H,E**) and Masson’s Trichrome. All micrographs show teratomas formed by mouse ESCs. Scale bars = 500 μm (far left panel only) and 100 μm. See also Figure S2.

Considering the developmental origins of MSCs, we examined expression of markers associated with early mesendoderm/hemangioblasts that have also been identified during stage-specific mesoderm induction from ESCs^16^,^17^. Using QPCR we found that levels of *GSC* (Goosecoid), *KDR* (also known as *FLK1*), *MIXL1*, *Brachyury* (also known as T) and *CXCR4* were increased significantly in 3D MSCs compared to the original 2D MSCs, whilst expression of CXCL12 was significantly decreased over the same time period (Fig. 2a). Using immunocytochemistry, we demonstrated that 2D MSCs were negative for Brachyury and CXCR4 protein whilst in 3D MSCs, both these markers were expressed throughout the condensate (Fig. 2b and Supplementary Fig. S2 for isotype controls). By image analysis using stringent thresholding and a nuclear counterstain for cell identification, we identified nuclear Brachyury immunopositivity evenly distributed throughout the spheroid (Supplementary Fig. S2). We next examined the tissue-generating capacity of 3D MSCs *in vivo.* When 3D MSCs were implanted into immunocompromised mice, they remained small and generated highly organized, segregated mesodermal structures including muscle, cartilage, adipose and connective tissue (Fig. 2c). This contrasted sharply with the implantation of mouse ESCs, which underwent uncontrolled proliferation leading to the formation of large, typically disorganized teratomas containing derivatives of all three germ layers, with limited, dispersed and unstructured mesodermal tissues (Fig. 2d). 2D MSCs were without effect and could not be retrieved. As 3D MSCs do not proliferate and rapidly redifferentiate when re-plated onto plastic, we trialed different non-adherent culture conditions to test growth properties whilst maintaining a dedifferentiated cell phenotype. We found that 3D MSCs could be disaggregated into small cell clumps and grown in semi-solid medium (in this case containing 1% methyl cellulose) with reduced serum content (5%) to limit the availability of differentiation-inducing cues. The disaggregated cells were able to expand as compact colonies, to retain diminished cell size and elevated expression of dedifferentiation markers compared to conventionally cultured 2D MSCs (Supplementary Fig. S2). 2D MSCs failed to survive when we attempted to culture them in identical non-adherent semi-solid medium conditions.

### Metabolic Transition in 3D condensates

**Figure 3:**
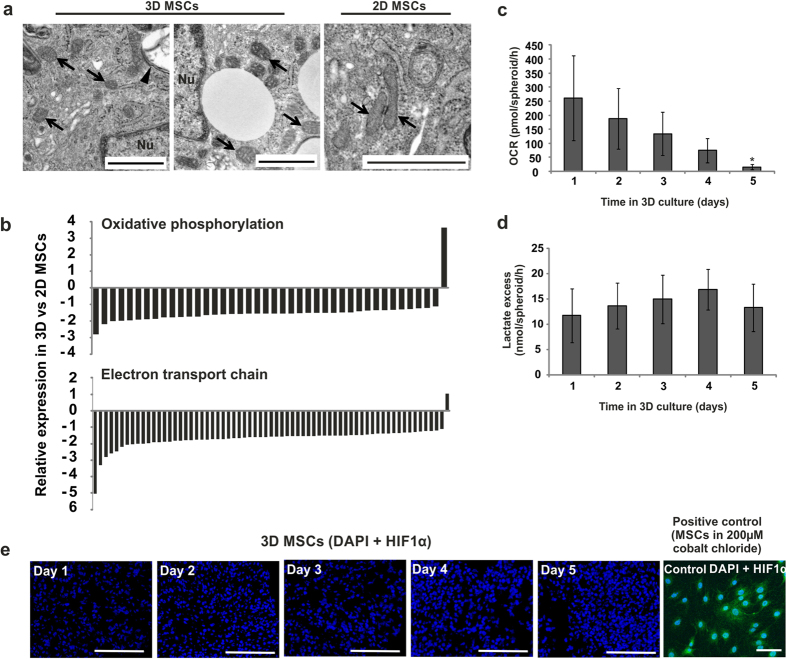
Metabolic transition in 3D MSCs. **(a**) Ultrastructural analyses by TEM revealed the presence of small, rounded mitochondria in 3D MSCs, and tubular mitochondria in 2D MSCs (arrows; arrowhead = double-membrane autophagic vesicle, Nu = nucleus, scale bar = 1 μm). (**b**) Expression levels of genesets associated with the electron transport chain pathway and oxidative phosphorylation pathway in 3D MSCs compared to 2D MSCs. Bars indicate expression levels for individual genes assigned to each pathway, for full gene lists see Tables S1 and S2. (**c**) Assay of cellular metabolic activity to determine oxygen consumption rate (OCR) of 3D MSCs over 5 days in culture. Data represent three separate experimental donors and are shown as mean values ± SEM, * = p < 0.05. (**d**) Analysis of excess lactate production by 3D MSCs over 5 days in culture. Data represent three separate experimental donors and are shown as mean values ± SEM. (**e**) Immunofluorescent staining (green fluorescence) of HIF1α in 3D MSCs between days 1 to 5 of culture (positive controls for hypoxia = MSCs cultured in 200 μM cobalt chloride, scale bars = 100 μm). Nuclei identified by DAPI staining (blue fluorescence). See also Figure S3 and Tables S1, S2.

In mechanistic analyses, we identified that dedifferentiation in 3D condensates was associated with a decrease in mitochondrial activity. By transmission electron microscopy (TEM), we observed a regression to rounded, immature mitochondria, similar to those observed in embryonic blastomeres and human ESCs^18^, and distinct from the tubular, elongated mitochondria observed in 2D MSCs (Fig. 3a). This was accompanied by widespread loss of expression of genes associated with electron transport chain function and oxidative phosphorylation (Fig. 3b, Supplementary Tables S1 and S2 and Supplementary Fig. S3), a feature of more primitive cell types including ESCs^19^,^20^. These changes corresponded with a shift from oxidative to ‘anaerobic-type’ metabolism, evidenced by decreased basal oxygen consumption and maintained lactate excess in 3D MSCs compared to 2D MSCs (Fig. 3c,d). This occurred without concurrent increases in HIF1α expression (Fig. 3e); confirming metabolic transition had taken place that was not induced by a hypoxic challenge. Glycolytic production of lactate in the presence of oxygen (aerobic glycolysis) has been confirmed in cells induced to dedifferentiate towards pluripotency^18^. Similarly, early blastemal cells have been reported to switch to aerobic glycolytic metabolism^21^.

### Autophagy accompanies cytoplasmic remodeling and dedifferentiation in 3D condensates

**Figure 4:**
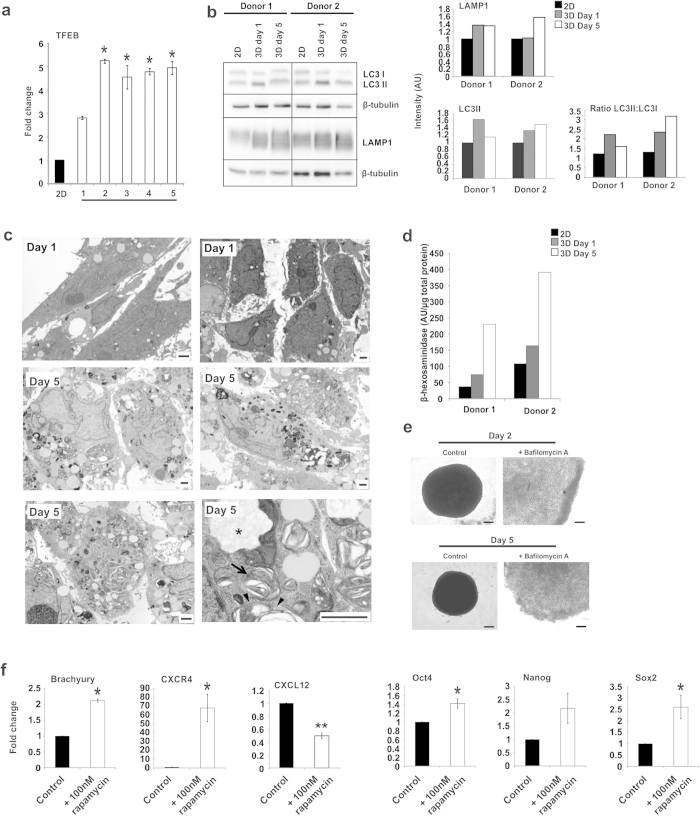
Analysis of Autophagy in 3D MSCs. (**a**) Analysis of *TFEB* expression in 3D MSCs versus 2D MSCs by QPCR. Data represent two separate experimental donors (n = 2) and are shown as mean values ± SEM, * = p < 0.05. (**b**) Western blot analysis of LAMP1 and LC3I and LC3II expression by 2D MSCs and 3D MSCs at days 1 and 5 of culture in two donors. Graphs show densitometric analysis of band intensity, normalized to β-tubulin expression of the individual donors. (**c**) TEM analysis of 3D MSCs on day 1 and day 5 of culture. At higher magnification (lower right panel) multilamellar bodies (arrows) and double membrane-bound vesicles (arrowheads) were observed. Asterisks identify vacuoles with presumptive degraded cell material (Scale bars = 1 μm). (**d**) Activity of lysosomal β-hexosaminidase in 2D MSCs and 3D MSCs (at days 1 and 5). Data shows β-hexosaminidase activity levels in two individual donors. Average values shown from two technical replicates. (**e**) Brightfield images of 3D MSC controls and 3D MSCs treated with Bafilomycin A on days 2 and 5 of culture, scale bar = 100 μm. (**f**) QPCR analysis of the effect of rapamycin on expression of *Brachyury, CXCR4, CXCL12, Oct4, Nanog* and *Sox,* in 2D MSCs. Data represent two separate experimental donors (n = 2) and are shown as mean values ± SEM, * = p < 0.05, ** = p < 0.01.

We hypothesized that the observed changes in cell behaviour during 3D condensation, including reduced cell size and metabolic transition, were mediated by enhanced autophagy flux. (Macro)autophagy is a physiological survival response that removes dysfunctional organelles and protein aggregates to maintain cytoplasmic quality and promote cell survival under stress/starvation. The removal of aged mitochondria can also reduce the leakage of damaging reactive oxygen species and toxic mitochondrial proteins, but will lead to a decrease in overall oxidative capacity. For dedifferentiation to occur, particularly for example in iPSC reprogramming, somatic cell restructuring, cytoplasmic clearance and a metabolic switch to glycolysis must be achieved to convert relatively large organelle-rich, phenotypically mature cell types to organelle-poor primitive cells and to activate and/or enable primitive function^20^. Interestingly, it has recently been demonstrated that autophagy is required early during iPSC reprogramming^22^, though in this case induced by ectopic Sox2 expression. Here, we determined expression of the transcription factor EB (TFEB), which coordinates lysosomal biogenesis and the expression of autophagy genes^23^,^24^. Compared to their originating 2D MSC population, we observed significantly increased mRNA levels of *TFEB* from day 2 of 3D MSC culture, which was maintained through to day 5 by QPCR (Fig. 4a). We also identified elevated expression of lysosome associated membrane protein 1 (LAMP1) in 3D MSCs, compared to 2D MSC controls, by western blot analysis, with levels increasing from day 1 and maintained through to day 5 of 3D culture (Fig. 4b). Similarly, during 3D condensation, we observed elevated expression of LC3II and an increase in the ratio of LC3II:LC3I, indicating a reduction in cytoplasmic LC3 and an increase in the autophagosome-incorporated form (Fig. 4b). Using TEM, we identified numerous vacuolar structures with heterogeneous content, including widespread electron-dense vesicles, multilamellar bodies and double membrane-limited structures; further evidence of extensive cytoplasmic remodeling and autophagy in 3D MSCs, which increased from day 1 to 5 (Fig. 4c). We also observed a marked increase in levels of the lysosomal enzyme β-hexosaminidase in 3D MSCs, indicating an increase in lysosome numbers compared to 2D MSCs (Fig. 4d). Exposure of 3D MSCs to the autophagy inhibitor Bafilomycin A was sufficient to inhibit the process of spheroid aggregation and condensation (Fig. 4e), suggesting a fundamental requirement for a functional autophagy response in this process. It was also possible to enhance expression of early mesoderm factors in 2D MSCs by inducing autophagy. Exposure to rapamycin (100 nM), an exogenous autophagy stimulator, was sufficient to increase mRNA expression of CXCR4, Brachyury, Oct4, Nanog and Sox2 in conventionally cultured 2D MSCs compared to untreated controls. Rapamycin treatment also resulted in a modest reduction in expression of CXCL12 in 2D MSCs, mimicking the expression changes observed in 3D condensates (Fig. 4f). Collectively, these data demonstrate a functional relationship between an activated autophagy response and the induction of primitive dedifferentiated features in human mesenchymal cells.

## Discussion

By attempting to mimic blastemal mesenchymal condensation using 3D culture systems, we have identified the intrinsic capacity of (aged) human cells to undergo efficient dedifferentiation to a primitive developmental stage from which they can redifferentiate to form organized tissues *in vivo.* The dedifferentiated cells are characterized by low mRNA expression of *Oct4, Nanog* and *Sox2* with high levels of early mesendoderm markers. Co-expression of both pluripotent and early mesendoderm transcripts has been observed during an early post-pluripotent stage of mesoderm induction from human ESCs^17^, suggesting that 3D MSCs could occupy a similar developmental position and broadly equivalent to blastemal cells^12^,^13^. The significance of pluripotent/reprogramming factor expression in regenerating blastemas is not clear and these factors have not been analysed in depth during mammalian dedifferentiation. However, elevated Oct4 and Nanog expression is associated with dedifferentiating β-cells as a consequence of diabetic β-cell failure in mice^25^. In addition, elevated expression of Oct4, Nanog and Sox2 are linked to tumour cell dedifferentiation in a range of different cancers^26^,^27^,^28^. Levels of *Oct4*, *Nanog* and *Sox2* in 3D MSCs are significantly lower than ESCs and 3D MSCs do not reach pluripotency but appear to undergo lineage-restricted dedifferentiation. This is demonstrated most clearly by the switch in the CXCL12/CXCR4 signalling axis, from CXCL12^High^ CXCR4^–^ cells, typical of the stromal phenotype, to a CXCL12^Low^ CXCR^+^ cell population. It should also be noted that we used MSCs isolated from aged donors undergoing orthopaedic surgery, which demonstrates the dedifferentiation capacity of MSCs from a relevant patient group. Although MSCs can show age-related variations in behavior^29^, we anticipate that dedifferentiation of MSCs from younger donors and even other stromal cell types could be similarly achieved.

For dedifferentiation to occur, the cytoplasmic complexity of committed cells must be restructured. Controlled autophagy, balanced towards its pro-survival/anti-aging effects, offers a biological mechanism to achieve this and recycle metabolites to fuel the regenerative process; similar to the essential role autophagy plays in early embryogenesis^30^. Autophagy is also a feature of iPSC generation by nuclear manipulation. The efficiency of factor-based cellular reprogramming is limited by stress-induced senescence/apoptosis and cell cycle arrest, which may be offset to some extent by an appropriate autophagy flux^31^. A varied autophagic response, caused by the stresses of factor-based reprogramming, may in part underlie its stochastic nature and recent work has shown that transient autophagy occurs during iPSC generation, although here initiated by ectopic Sox2 expression^22^. The mTOR inhibitor rapamycin significantly increases the efficiency of iPSC generation^32^ suggestive of a link between autophagy activation and dedifferentiation. Elevated autophagy was a notable feature of our mesenchymal condensates and autophagy inhibition through treatment with the lysosomal H^+^ ATPase inhibitor, Bafilomycin A, prevented their formation. In addition, we found that treatment of normal 2D-adherent MSCs with rapamycin resulted in a modest, but significant enhancement in expression of pluripotency and early hemangioblast/mesendodermal factors, providing further evidence of autophagy-driven dedifferentiation. Our optimised 3D culture model will provide highly specific environmental conditions, which we propose induce balanced autophagy, reduce cell size and complexity, restrict growth/hypertrophy and cause bioenergetic shift to enable dedifferentiation. Recapitulating 3D dedifferentiating conditions in standard 2D culture will be challenging using chemical modifiers of autophagy alone, such as rapamycin. Autophagy can be initiated through numerous mTOR-dependent and mTOR-independent mechanisms, including cAMP/PLC, IP3/inositol, calcium channel/Ca^2+^/calpain and Bcl2/Beclin, which can be targeted with chemical modifiers^33^. Therefore, we anticipate that dedifferentiation conditions may be met by autophagy pathway manipulation in 2D cultures once the precise mechanisms have been identified. The role of autophagy in blastema formation has not been extensively explored, however it is interesting to note that the tissue regenerative capacity of hydra is dependent on a finely balanced autophagy response^34^ and recent work has demonstrated the requirement for autophagy during fin regeneration in zebrafish^35^. Our findings now show that it is possible to mimic many features of blastema formation using human MSC condensates. This study also highlights balanced autophagy as a potential driving mechanism for dedifferentiation and the acquisition of primitive features in human cells, which will impact on our understanding of tissue dedifferentiation and regeneration.

## Experimental Procedures

### Cell Culture

Human mesenchymal stromal cells (MSCs) were isolated from femoral heads/knee bone explants from joint replacement patients as previously described^36^, under the approval of the York Local Research Ethical Committee. Informed consent was obtained from all patients. All experimental protocols were carried out in accordance with the University of York Department of Biology Ethics Committee guidelines and were approved by the South Humber Local Research Ethics Committee.

MSCs were cultured as adherent monolayers in Dulbecco’s modified Eagle’s medium (DMEM, high glucose) supplemented with 15% fetal bovine serum (FBS) (Invitrogen). MSCs were positive for stromal markers CD29, CD44, CD73, CD90, CD105, CD166, and negative for the hematopoeitic markers CD34 and CD45 (data not shown). For 3D culture, monolayer (2D) MSCs were grown to approximately 90% confluence, trypsinized and resuspended in 3D medium; DMEM (high glucose), supplemented with 15% FBS and 0.25% methyl cellulose (Sigma).

### 3D Growth Conditions

MSCs were seeded into U-bottomed 96-well plates with 30,000, 60,000, or 120,000 cells per well in medium containing 0.25% methyl cellulose. Cells self-aggregated to form 3D MSCs within 5 hours. For studies requiring disaggregation, 3D MSCs were collected, washed in PBS and then resuspended in Liberase TL working solution (Liberase TL (Roche) 1.3 Wunsch units per ml in PBS). 3D MSCs were incubated on an orbital shaker at 150 rpm for 20 minutes at 37 °C and then disaggregated to a single cell suspension by pipetting, before reseeding onto tissue culture plastic in 2D MSC media. Cell areas of *in vitro*-aged MSCs, before and after 3D culture, were calculated using Image J.

### EdU proliferation assay

Proliferation was assessed using the Click-IT EdU Alexa 488 imaging kit (Life Technologies). 2D and 3D MSCs were cultured as described above for 7 days, in media supplemented with 10 μM EdU, medium was refreshed daily. 3D samples were snap frozen and sectioned on day 7. 2D and 3D samples were stained according to manufacturer’s instructions, with Hoechst 33342 counterstaining. Imaging was performed using the LSM510 confocal imaging system.

### RNA extraction

For RNA extraction, 2D MSCs were trypsinized, centrifuged at 1200 rpm for 5 minutes, and then re-suspended in lysis buffer (Nucleospin RNA II kit) with 1% β-mercaptoethanol. 3D MSC spheroids were collected into 1.5ml tubes, washed with PBS, re-suspended in lysis buffer as above, before homogenisation with a hand-held tissue micro-homogeniser for 5 seconds. H9 ESC colonies were trypsinized using recombinant trypsin (TrypLE Invitrogen) and resuspended in lysis buffer as above. RNA was then extracted using Nucleospin RNA II columns (Macherey Nagel) following manufacturer’s instructions.

### Quantitative polymerase chain reaction (QPCR)

cDNA was synthesized from 1 μg mRNA and QPCR was performed with Power SYBR Green PCR Master Mix (Applied Biosystems) using the Applied Biosystems 7300 Real Time PCR System (50 °C for 2 minutes, 95 °C for 10 minutes followed by 40 cycles of 95 °C for 15 seconds, 60 °C for 1 minute). Fold changes were calculated as follows: Delta (D) C_t_ values were calculated by normalising C_t_ values for target genes to average C_t_ values for GAPDH, which was selected as an appropriate housekeeping gene from three others (β-actin, B2M, RPS27a). DDC_t_ values were then calculated between control conditions and experimental conditions. Fold changes were calculated as 2(-DDCt), relative to expression in the originating 2D MSC population. Statistical analyses were performed using Sigmaplot software. Primer sequences are provided in Supplementary Information.

### Statistical Analysis

QPCR data were analyzed using Kruskal Wallis One Way Analysis of variance on ranks (with Tukey test for pairwise multiple comparison procedures) or Mann-Whitney Rank Sum Test. An astericks indicates a statistically significant difference between the value observed in a particular sample and the value in the originating 2D MSC population. The number of donors examined in each experiment are given in each figure legend as (n = x), 3 technical replicates were performed for each donor.

### Immunocytochemistry

For 2D cultures, the cells were seeded on coverslips and allowed to adhere overnight. 3D MSCs were embedded in OCT Tissue Tek and cryosectioned at 5–7 μm. Sections and coverslips were fixed in 4% paraformaldehyde for 10 minutes followed by overnight immunostaining for Ki67 (4 μg/ml, Abcam). HIF1α (5 μg/ml, Abcam), Brachyury (15 μg/ml, R and D Systems) or CXCR4 (10 μg/ml, Chemicon (Millipore)) according to the manufacturers’ instructions with a DAPI nuclear counterstain. Exposure to 200 μM cobalt chloride for 4 hours was used as a positive control for hypoxia (HIF1α). Samples were then incubated with fluorescently-conjugated secondary antibodies (Alexafluor 488 rabbit anti-mouse 1:500; Sigma rabbit anti-goat Cy3 1:400; Sigma goat anti-rabbit Cy3 1:400), before nuclear counterstaining with 4′,6-diamidino-2-phenylindole (DAPI). Slides were imaged using the LSM510 confocal imaging system. LSM images of Brachyury immunostaining were analyzed using Volocity image analysis software to calculate % Brachyury-positive nuclei against DAPI staining. Volocity was also used to identify positions of the lowest and highest Brachyury immunofluorescent intensities in 48 of 761 cells each to determine distribution of positivity.

### *In vivo* assays

The *in vivo* study was performed independently by Reinnervate Ltd (Co. Durham, UK). Mouse ESCs (E14) included as positive controls were cultured and prepared by Reinnervate Ltd. All work was carried out in accordance with Home Office ethical guidelines. All experimental protocols were approved by the Home Office project licence of Reinnervate Ltd. 3D MSCs, initiated at 60,000 cells and cultured for five days and 2D MSCs cultured as monolayers were injected subcutaneously into adult male nude (Nu/Nu) mice. Mouse ESCs (E14) included as positive controls. 2D MSCs and ESCs were trypsinised, counted and 6 × 10^5^ cells were injected into the mice using a matrigel carrier. For the 3D MSCs, 10 spheroids were injected per mouse in matrigel. Mice were sacrificed after 12 weeks in accordance to ethical guidelines. Samples were recovered and fixed immediately for assessment of teratoma formation. Sections of 5 μm thickness were cut using a Leica RM2165 rotary microtome, collected onto slides and dried at 40°C in an oven prior to being stained using hematoxylin and eosin and Masson’s Trichrome. MSCs from 2 different primary donors were used, and for each donor, samples were injected into 3 mice. Tissue masses were recovered from 2/3 mice injected with 3D MSCs from both donors (total number of 4/6). 3 mice were injected with control mouse ESCs, and teratomas were recovered from 100% of injected mice.

### Transmission electron microscopy (TEM)

2D MSCs were trypsinized and centrifuged to a cell pellet. 2D and 3D samples were fixed in 8% formaldehyde, 5% glutaraldehyde in 100 mM phosphate buffer mixed 50/50 with 2D or 3D MSC media for 10 minutes, followed by fixing 4% formaldehyde, 2.5% glutaraldehyde in 100 mM phosphate buffer, pH7.2 for 30 minutes at room temperature. Osmium tetroxide (OsO_4_) was used as a secondary fix, before dehydration through an ethanol series followed by epoxypropane. Finally samples were infiltrated and embedded in Epon Araldite. 70 nm sections were cut from the polymerised sample blocks and then stained with saturated uranyl acetate in 50% ethanol and Reynold’s lead citrate. The sections were imaged on a FEI Tecnai G transmission electron microscope. Enhanced contrast was used to optimize mitochondrial imaging (in Fig. 3A). Following primary fixation, samples were treated as follows: 100mM phosphate buffer 2 × 20 minutes, 1% tannic acid in 100 mM phosphate buffer for 10 minutes, followed by 100mM phosphate buffer 2 × 20 minutes. Samples were then placed on ice in 0.5% OsO_4_ for one hour followed by washing with water for 2 × 20 minutes before being placed in the dark in 1% uranyl acetate in water for one hour, washed in water for 20 minutes, and then left overnight in water at 4 °C. The next day, samples were passed through an acetone series: 25% acetone, 20 minutes; 50% acetone, 20 minutes; 70% acetone, 20 minutes; 90% acetone, 20 minutes; 100% acetone 2 × 25 minutes, followed by 25% Spurr (R): 75% acetone 30 minutes; 50% Spurr (R): 50% acetone 30 minutes; 75% Spurr (R): 25% acetone 30 minutes; 100% Spurr (R) 2 changes, 2 hours then 30 minutes each. Polymerisation was at 70 °C overnight.

### Metabolic measurements

Oxygen consumption by 3D MSCs was measured using the BD Biosci-ences Oxygen Biosensor System (OBS). Briefly, two MSC spheroids were cultured in triplicate wells of the OBS plates in 50 μl of culture medium, alongside blank wells containing medium alone. Kinetic measurements were taken every 20 seconds for 30 minutes. Raw fluorescence data was corrected against a pre-blank reading and converted to oxygen concentration in accordance with manufacturer’s guidelines. The oxygen consumption rate (OCR) was calculated as the gradient of change in oxygen concentration over time in nmol/spheroid/hr. Glucose depletion and lactate release into the spent medium collected at the end of the oxygen assay was determined by using enzyme linked fluorescent assays as previously described^37^.

### Western blot analysis

Monolayer MSCs were trypsinized, centrifuged and re-suspended in RIPA buffer (Thermo Scientific) containing 0.5% protease inhibitor cocktail set III (Calbiochem) and 100 μM Na_3_VO_4_ (Sigma). 3D MSC spheroids were homogenized in RIPA buffer as above. For LAMP1 immunoblotting, 20 μg of total protein was loaded onto a 10% SDS polyacrylamide gel, electrophoresed, transferred to a PVDF membrane and probed with an anti-LAMP1 antibody (Developmental Studies Hybridoma Bank, University of Iowa). For LC3 immunoblotting, 20 μg of total protein was loaded onto a 15% SDS polyacrylamide gel, which following electrophoresis, were transferred to a PVDF membrane and probed with an anti-LC3 antibody (Nanotools). All blots were also probed with an anti-β-Tubulin antibody (Sigma) to act as a loading control. Detection was performed by enhanced chemiluminescence (ECL) following manufacturer’s instructions (Promega) and intensities quantified using Image J analysis software.

### β-hexosaminidase quantification

100 μl substrate solution (100 mM Citrate buffer pH 5.0, 0.5 mM 4-methylumbelliferyl-2-acetamido-2-deoxy-beta-D-glucopyranoside, 0.27M Sucrose) was added to 5 μl total protein (in duplicate) and incubated for 3 minutes. The reaction was stopped with 1 M Na_2_CO_3_, after which a fluorimeter reading was taken (excitation 360 nm, emission 445 nm) and equated to the protein concentration of each sample.

### Bafilomycin treatment of 3D MSCs

3D MSC spheroids were seeded and cultured for 5 days as described in the main text Experimental Procedures. All 3D MSC medium was refreshed daily. Treated spheroids were fed with media supplemented with 100 nM Bafilomycin A (Sigma) in DMSO. Samples were imaged using light microscopy on days 2 and 5. 3D MSCs fed with media supplemented with DMSO alone were indistinguishable from control spheroids.

### Rapamycin treatment of 2D MSCs

2D MSCS were seeded and cultured as described in the main text Experimental Procedures. Treated samples were incubated in media supplemented with 100 nM Rapamycin (Fisher Scientific Ltd) for 24 hours. RNA was then extracted from samples as described above.

### Genome wide expression analysis

Microarray data can be found on ArrayExpress E-MTAB-3795. For methods please see Supplementary Information.

## Additional Information

**How to cite this article**: Pennock, R. *et al.* Human cell dedifferentiation in mesenchymal condensates through controlled autophagy. *Sci. Rep.* **5**, 13113; doi: 10.1038/srep13113 (2015).

## Supplementary Material

Supplementary Information
