# Supplementary material for: Human cell dedifferentiation in mesenchymal condensates through controlled autophagy
Source: Sci Rep. 2015 Aug 20;5:13113. doi: 10.1038/srep13113 (PMC4542335; doi:10.1038/srep13113)
Supplement: Supplementary Information [file srep13113-s1.pdf]

Human cell dedifferentiation in mesenchymal condensates through controlled autophagy

AUTHORS

Rebecca Pennock, Elen Bray, Paul Pryor, Sally James, Paul McKeegan, Roger Sturmey and Paul Genever

SUPPLEMENTARY FIGURES

Supplementary Figure S1.

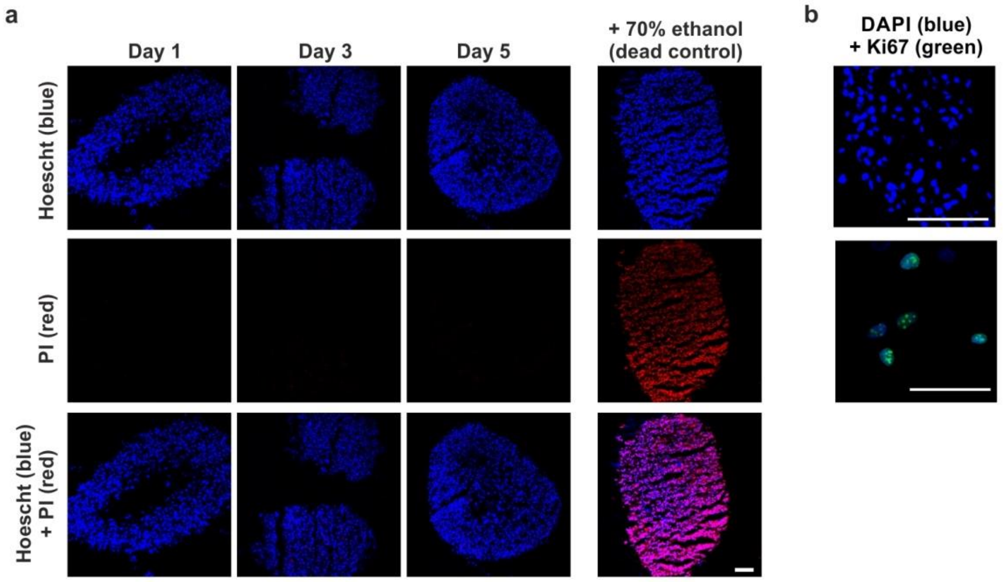

Supplementary Figure S2.

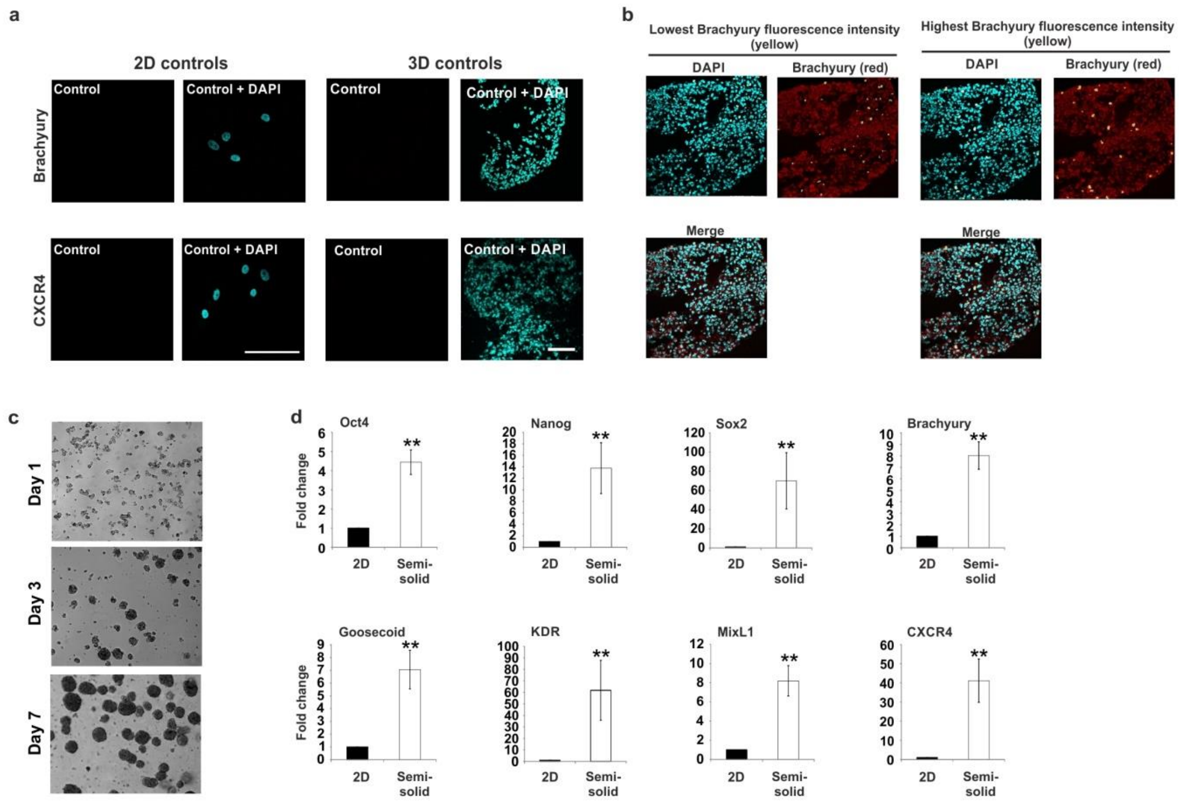

Supplementary Figure S3.

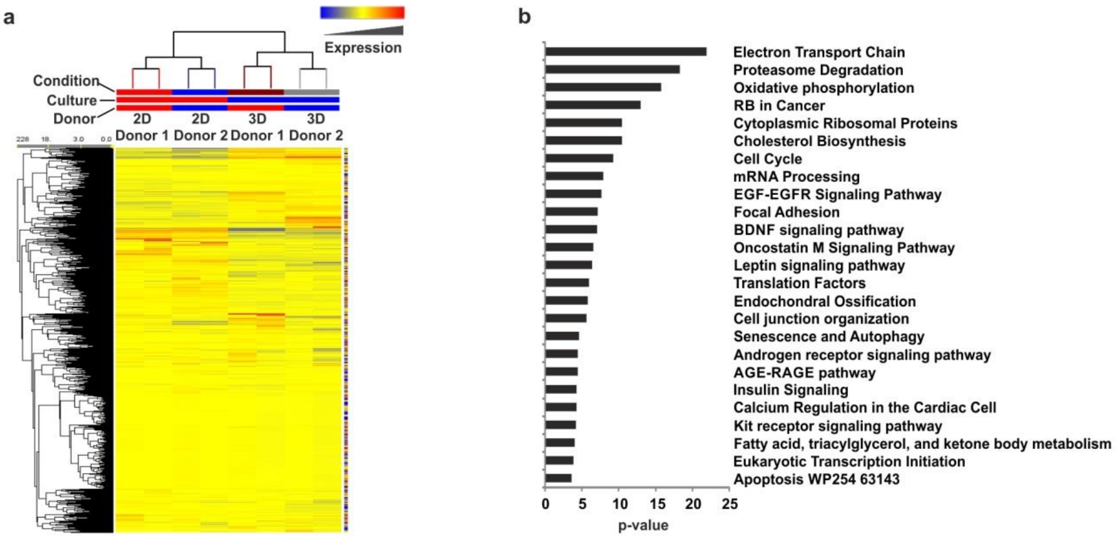

SUPPLEMENTARY FIGURE LEGENDS

Supplementary Figure S1. Cell viability and proliferation in 3D MSCs

(a) Staining of 3D MSCs with PI and Hoechst 33342. Representative images of Day 1, Day 3 and Day 5 3D MSCs and killed control 3D MSCs + 70% ethanol are shown (scale bar = 100µm).

(b) Immunofluorescent detection of Ki67 in 3D MSCs (2D MSCs are shown as a positive control), scale bar = 50µm

Supplementary Figure S2. Analysis of dedifferentiation status of 3D MSCs

(a) Brachyury and CXCR4 isotype controls in 2D MSCs and 3D MSCs (scale bar = 100µm). Nuclei identified by DAPI staining (blue fluorescence).

(b) Image analysis of Brachyury-immunopositive nuclei in 3D MSCs, showing lowest- (yellow, left panel) and highest- (yellow, right panel) expressing cells, distributed evenly throughout the spheroid. DAPI nuclear staining shown as blue fluorescence.

(c) Cell growth assays following disaggregation of 3D MSCs and re-suspension in semi-solid media (scale bar = 100µm).

(d) QPCR analysis of pluripotency and mesendodermal marker expression following disaggregation of 3D MSCs and re-suspension in semi-solid media. Data represent two separate experimental donors and are shown as mean values ± SEM, ** = $p<0.01$.

Supplementary Figure S3. Transcriptomic analyses of differentially expressed pathways in 3D MSCs versus 2D MSCs

(a) Clustering of 2D MSCs and 3D MSCs from 2 different donors according to culture condition (2D or 3D) and donor.

(b) The top 25 significantly differentially expressed pathways in 3D MSCs versus 2D MSCs from gene pathway analysis.

SUPPLEMENTARY TABLES

Gene name	Fold change	Gene name	Fold change
ND5	-6.1126	ATP5S	-1.57138
ND4L	-5.05674	AQDQ	-1.56911
ND3	-4.04979	ATP5G3	-1.5637
ND4	-3.03716	ATP5L	-1.54443
ATP5G1	-2.84382	ATP5G2	-1.53316
NDUFS8	-2.20634	ATP5O	-1.53188
B18	-2.03715	NDUFA11	-1.52613
NDUFB2	-2.01686	B14	-1.52465
ATP5J2	-1.98975	CI-SGDH	-1.50213
ATP6	-1.9447	NDUFA2	-1.49991
NDUFA4	-1.93893	ATP5J	-1.438
B14.5a	-1.93	NDUFS5	-1.39763
ATP5F1	-1.89167	ATP5E	-1.37441
ATP5D	-1.8098	ATP6AP1	-1.37354
NDUFS6	-1.79843	CI-51kD	-1.36005
ATP5H	-1.77936	CI-75Kd	-1.34605
KFYI	-1.76233	ATP6AP2	-1.34393
ATP5A1	-1.75855	B22	-1.33539
ATP5I	-1.75112	B14.5b	-1.2961
B9	-1.66852	NDUFS3	-1.26757
GZMB	-1.65155	NDUFV2	-1.25547
B17	-1.63827	NDUFS7	-1.2454
CI-SGDH	-1.62896	CI-42KD	-1.1436
ASHI	-1.60262	ND2	-1.091
NDUFV3	-1.59722	B13	-1.086
NDUFA8	-1.5948	B15	1.046901
ATP5B	-1.58734	ND1	1.089706
NDUFB10	-1.57776	NDUFS2	1.108659
NDUFA9	-1.575	NUOMS	3.654102
FASN2A	-1.57454	ND6	4.23435

Supplementary Table S1. Fold change expression of genes assigned to the mitochondrial oxidative phosphorylation pathway (using Genespring v12.1) in 3D MSCs vs 2D MSCs.

Gene name	Fold change	Gene name	Fold change
ND4L	-5.05674	NDUFA8	-1.5948
SLC25A4	-3.34134	ATP5B	-1.58734
ATP5G1	-2.84382	NDUFB10	-1.57776
COX7A1	-2.61804	NDUFA9	-1.575
ATPIF1	-2.48431	NDUFAB1	-1.57454
NDUFS8	-2.20634	ATP5S	-1.57138
SLC25A5	-2.09295	NDUFS4	-1.56911
COX8A	-2.07	ATP5G3	-1.5637
NDUFB7	-2.03715	ATP5L	-1.54443
NDUFB2	-2.01686	COX6B1	-1.53532
ATP5J2	-1.98975	ATP5G2	-1.53316
NDUFA4	-1.93893	ATP5O	-1.53188
NDUFA7	-1.93	COX7B	-1.52843
COX5A	-1.89533	NDUFA6	-1.52465
ATP5F1	-1.89167	COX7C	-1.51127
UCRC	-1.84434	NDUFB5	-1.50213
DAP13	-1.84087	NDUFA2	-1.49991
ATP5D	-1.8098	COX5B	-1.4669
UQCRFS1	-1.80153	ATP5J	-1.438
NDUFS6	-1.79843	SURF1	-1.41699
ATP5H	-1.77936	NDUFA1	-1.40509
NDUFB3	-1.77505	NDUFS5	-1.39763
NDUFC1	-1.76233	SCO1	-1.3874
ATP5A1	-1.75855	ATP5E	-1.37441
COX6A1	-1.75063	NDUFS1	-1.34605
QP-C	-1.70969	NDUFB9	-1.33539
ATP5C1	-1.69572	UQCRB	-1.3215
SDHB	-1.68605	NDUFC2	-1.2961
NDUFA3	-1.66852	NDUFS3	-1.26757
NDUFB6	-1.63827	NDUFS7	-1.2454
NDUFB1	-1.62896	UQCRC1	-1.23871
SDHC	-1.6136	NDUFA10	-1.1436
UQCRH	-1.61309	COX11	1.054393
NDUFB8	-1.60262		

Supplementary Table S2. Fold change expression of genes assigned to the electron transport chain pathway (using Genespring v12.1) in 3D MSCs vs 2D MSCs.

SUPPLEMENTARY METHODS

Assay of dead cell numbers in 3D MSCs

3D spheroids initiated from 60,000 MSCs were collected at days 1, 3, 5. A sample at each time point was incubated with 70% ethanol for 45 minutes, then rinsed in PBS and incubated with 1µg/ml PI for 4 hours. These samples served as dead controls. For the test samples at each time point, samples were rinsed in PBS and incubated with 1µg/ml PI for 4 hours. Samples were then isolated, snap frozen and sectioned. After sectioning, samples were stained with Hoechst 33342. Imaging was performed using the LSM510 confocal imaging system, all samples were imaged using identical imaging conditions.

Quantitative polymerase chain reaction (QPCR)

Gene	Primer sequences - forward and reverse (5'- 3')	GenBank accession number
GAPDH	TGCACCACCAACTGCTTAGC<br>GGCATGGACTGTGGTCATGAG	NM_002046
Oct4	CCCACACTGCAGCAGATCAG<br>CACACTCGGACCACATCCTTCT	NM_002701
Nanog	CCTCCATGGATCTGCTTATTCAG<br>TGCGACACTATTCTCTGCAGAAG	NM_024865
Sox2	GAGAACCCCAAGATGCACAAC<br>CGCTTAGCCTCGTCGATGA	NM_003106
TFEB	CCAGAAGCGAGAGCTCACAGAT<br>TGTGATTGTCTTTCTTCTGCCG	NM_007162
Brachyury (T)	GGGTCCACAGCGCATGAT<br>TGATAAGCAGTCACCGCTATGAA	NM_003181
Goosecoid	GATGCTGCCCTACATGAACGT<br>GACAGTGCAGCTGGTTGAGAAG	NM_173849
KDR	TGATGCCAGCAAATGGGAAT<br>CCACGCGCCAAGAGGCTTA	NM_002253
MIXL1	AAGCCCCAGCTGCCTGTT<br>CCCTCCAACCCCGTTTG	NM_031944
CXCR4	CGCCTGTTGGCTGCCTTA<br>ACCCTTGCTTGATGATTTCCA	NM_003467
CXCL12	CCGTCAGCCTGAGCTACAGAT<br>GACGTTGGCTCTGGCAACA	NM_199168.3

Table showing primer sequences used for quantitative polymerase chain reaction (QPCR).

Semi-solid expansion assay

For semi-solid expansion studies, MSCs were cultured as 60,000 cell spheroids for 5 days and disaggregated to small cell clumps as described above. Cell suspensions in DMEM, high glucose, were then mixed with semi-solid media (either medium 1: DMEM high glucose supplemented with 5% FBS and 1% methyl cellulose (final concentrations), or medium 2: ESC medium supplemented with 1% methyl cellulose (final concentration)). MSCs were cultured at 37°C, 5% CO2 for 7 days. For RNA extraction, MSC aggregates were isolated by washing with PBS and then pelleted by centrifugation, before RNA extraction was performed using Trizol following manufacturer's instructions (Gibco). cDNA synthesis and QPCR was performed as described above.

Global gene expression analysis

For global gene expression analyses, duplicate RNA samples from two donors, maintained for 5 days in 2D or 3D culture were harvested as previously described. RNA quality was assessed using the Agilent 2100 Bioanalyzer with RNA 6000 nano chips and 150ng high quality RNA per sample (with RNA integrity numbers of 9.8 or greater), spiked with control RNA species from the Agilent One color spike-in kit, were Cy3-labelled using the Agilent One Color Low Input Quick Amp labeling kit. 600ng Cy3-labelled RNA per sample was then hybridized onto an Agilent SurePrint G3 Human Gene Expression 8x60K v2 Microarray and washed as per the manufacturer's guidelines. Arrays were scanned using an Agilent DNA Microarray Scanner (G2565CA) with SureScan High-Resolution Technology. Data were analyzed using Genespring version 12.1 software (Agilent technologies), and all quality control checks met the standards required. This software was then used to draw clustered heatmaps of global expression data, and perform relevant statistical analyses. A paired sample t-test was employed, using the Benjamini - Hochberg

algorithm to control for the effects of multiple testing. This procedure allowed for the adjustment of the p-values and the lowering of the false discovery rate whilst keeping the sensitivity for true positive results. Genes with a minimum two-fold difference in expression level and an adjusted p-value of <0.05 were considered differentially expressed. Subsequent pathway analysis was performed on lists of genes differentially expressed in response to change in culture conditions, using the pathway analysis tool within Genespring v12.1 software.

Ethical Approvals

The *in vivo* study was performed independently by Reinnervate Ltd (Co. Durham, UK). Mouse ESCs (E14) included as positive controls were cultured and prepared by Reinnervate Ltd. All work was carried out in accordance with ethical guidelines under the Home Office project licence of Reinnervate Ltd. Human MSCs were obtained following full informed consent under approval of the York Local Research Ethical Committee.
